# Infertility service availability, geographic accessibility, and use: results from linked facility-female cross-sectional data in Burkina Faso, Côte d’Ivoire, Niger, and Uganda

**DOI:** 10.1186/s12913-026-15214-6

**Published:** 2026-08-04

**Authors:** Haley L. Thomas, Fredrick Makumbi, Philip Anglewicz, Simon P. S. Kibira, Caroline Moreau, Georges Guiella, Rosine Mosso, Suzanne O. Bell

**Affiliations:** 1 Department of Population Family and Reproductive Health, Johns Hopkins Bloomberg School of Public Health, Baltimore, MD USA; 2 Makerere University School of Public Health, Kampala, Uganda; 3 Soins Primaires et Prevention, CESP Centre for Research in Epidemiology and Population Health, U1018, Inserm, Villejuif, France; 4 Institut Supérieur des Sciences de la Population, (ISSP/Université Joseph Ki-Zerbo), Ouagadougou, Burkina Faso Burkina Faso; 5 Ecole Nationale Superieure de Statistique et Appliquee d’Abidjan (ENSEA), Abidjan, Côte d’Ivoire

**Keywords:** Infertility, Sub-Saharan Africa, Survey, Health facilities, Sexually transmitted infections, Care-seeking, Fertility care

## Abstract

**Background:**

Infertility affects millions of people globally, with the highest prevalence in Africa (16%). Despite strong evidence linking sexually transmitted infections (STIs) to infertility in sub-Saharan Africa, evidence on the availability of and accessibility to infertility-related services—including STI testing and broader infertility care—remains limited across the region. This study aims to: (1) assess availability by estimating the proportion of facilities offering STI testing and infertility services; (2) assess geographic accessibility by examining women’s proximity to service-offering facilities; and (3) assess use by evaluating whether geographic proximity is associated with biomedical care-seeking for difficulties conceiving.

**Methods:**

We used nationally representative cross-sectional survey data from women aged 15–49 and the health facilities that serve them in Burkina Faso, Côte d’Ivoire, Niger, and Uganda (2021–2024). We estimated the proportion of facilities offering STI testing and infertility services, the latter being a broad facility-reported measure of the availability of any service, without specification on the type of service. Using geospatial analysis, we linked facility data to women’s residences and calculated the proportion of women living within 5 and 10 km of STI testing and infertility services, respectively. We fit multivariable logistic regression models to examine associations between women’s characteristics and geographic access to services, and between geographic access and women’s biomedical care-seeking.

**Results:**

STI testing was widely available across countries (68.5–92.6% of facilities), while infertility services were limited (28.4–44.0%), except in Burkina Faso (78.3%). Women with higher education, greater wealth, and urban residence had higher odds of geographic access to services. In Burkina Faso and Niger, women living within 5 km of STI testing facilities had increased odds of biomedical care-seeking (aOR 2.94 and aOR 7.19, respectively), and in Niger, women within 10 km of infertility services had increased odds of biomedical care-seeking (aOR 8.46).

**Conclusions:**

Infertility service availability and accessibility remain limited, and women’s proximity to services is associated with biomedical care-seeking, underscoring the importance of service expansion. Governments and NGOs should prioritize investments in STI prevention and treatment and, where feasible, more advanced infertility evaluation and treatment, including referral pathways to higher-level care, to address the region’s unmet need for infertility services.

**Supplementary Information:**

The online version contains supplementary material available at https://doi.org/10.1186/s12913-026-15214-6.

## Background

One in six people globally are affected by infertility—defined clinically as the inability to achieve a pregnancy after 12 months of regular unprotected sexual intercourse—with notable variation in prevalence by geographic region [1–3]. The Africa region is estimated to have the highest 12-month period prevalence of infertility in the world, at 16.4% [1]. Women and couples in this region experience severe psychological, physical, social, and financial consequences of infertility given the immense social pressure to reproduce [4, 5], making infertility a pressing public health issue. However, prevention and treatment remain challenging and understudied, particularly in sub-Saharan Africa, where infertility has historically not been a public health priority [6–9].

Infections, most commonly sexually transmitted infections (STIs), are a leading cause of infertility in sub-Saharan Africa [10–16]. Untreated STIs—particularly chlamydia and gonorrhea—can cause pelvic inflammatory disease, tubal scarring, and subsequent tubal factor infertility [16]. A multi-country investigation by the World Health Organization (WHO) found that 89% of African women with an infection-related infertility diagnosis had a history of STIs [17]. More recently, a 2024 study among women experiencing infertility in the Democratic Republic of the Congo found that those with *Chlamydia trachomatis* infection had 14 times the odds of tubal factor infertility than those without the infection [15]. Despite being preventable with timely and adequate treatment, existing evidence indicates that availability of and access to STI diagnostics and treatment remains limited in the region [18–21], contributing to disparities in access to STI care [22]. STI testing is the necessary first step in the infertility prevention pathway; therefore, the availability of and access to STI testing is a critical component of infertility prevention services. Timely identification and treatment of STIs represents the most feasible point of intervention in resource-constrained settings where advanced infertility treatment remains limited [16].

Other major causes of infertility include unsafe abortion practices, complications in childbirth, and non-STI-related male infertility [23]. Addressing these diverse causes of infertility requires a range of medical interventions with varying degrees of advanced training and/or equipment, from less complex procedures like ovulation induction and intrauterine insemination (IUI), to more advanced assisted reproductive technologies (ART) such as in vitro fertilization (IVF) and intracytoplasmic sperm injection (ICSI). These services are highly limited or unavailable for the majority of people in sub-Saharan Africa. Existing data indicate fewer than 250 assisted reproduction cycles per million population are provided every year [24] despite 1,500 cycles per million needed to meet the demand for ART [6, 25]. Existing services are often concentrated in capital cities and other major urban areas, making them geographically inaccessible to rural residents [6, 25]. Available services also often lack the medical sophistication needed to address the underlying issue of most clients or are financially inaccessible, leading many to seek traditional sources of care [6, 13, 23, 26–29]. Consequently, only those with substantial financial resources and/or proximity to specialized urban facilities may access biomedical fertility care, exacerbating existing healthcare inequities.

Significant gaps exist in our understanding of infertility care in sub-Saharan Africa. First, we have limited national information regarding the availability of services to prevent (via STI testing and treatment) and treat infertility in nearly all sub-Saharan African countries; we know of only one study from the Gambia [30]. Second, there is no comprehensive study examining disparities in individuals’ geographic access (i.e., proximity) to infertility care across the region, leaving critical questions about who can reasonably obtain these services. Third, we lack insights into how proximity to infertility services may influence their usage. Addressing these knowledge gaps is essential for developing targeted policies and interventions that promote equitable access to infertility care and support across diverse settings in sub-Saharan Africa.

Using nationally representative population-based data of reproductive-aged women and the facilities that serve them, we sought to understand infertility-related service availability, geographic accessibility, and use in four sub-Saharan African countries: Burkina Faso, Côte d’Ivoire, Niger, and Uganda. Specifically, we aimed to estimate the proportion of facilities offering STI testing and infertility services, to examine variation in geographic proximity to facilities offering these services by women’s sociodemographic and reproductive characteristics, and to evaluate whether geographic proximity to these services is related to women’s use of biomedical care for difficulties conceiving. Throughout this paper, we treat STI testing as an infertility prevention service, given this well-established pathway from untreated STIs to infertility, and ‘infertility services’ as a broad facility-reported category that may encompass counseling, evaluation, and treatment.

## Methods

Data for this study come from Performance Monitoring for Action (PMA), a data collection platform that administers national or sub-national surveys at the household, female, and health facility level in eight countries in sub-Saharan Africa and Asia [31]. Trained interviewers who reside in or near surveyed communities conduct face-to-face interviews using mobile phones with the Open Data Kit (ODK) software to record responses. Data analyses for this study utilized cross-sectional female and facility data from Burkina Faso, Côte d’Ivoire, Niger, and Uganda. These four countries were selected because comparable nationally representative female and health facility data from the PMA platform were available, and together they represent diverse geographic, epidemiologic, and health system contexts within sub-Saharan Africa. Additional details about the PMA project can be found at pmadata.org.

### Samples of women

PMA used a multi-stage stratified cluster design with probability proportional to size sampling of smaller geographic areas (enumeration areas or EAs) to obtain nationally representative samples of women aged 15–49 in each country. The sampling process included: (1) probability proportional to size selection of counties/regions, followed by urban-rural stratified selection of EAs within selected counties; (2) mapping and random selection of households within each EA; and (3) completion of a household roster to identify all women aged 15–49 for the female survey. In Burkina Faso, data were collected between December 2021 and February 2022 from 5,377 households (97% response rate) and 6,078 women aged 15–49 (93% response rate). Data were collected in Côte d’Ivoire from 3,679 households (96% response rate) and 3,634 women aged 15–49 (94% response rate) between November 2023 and February 2024. In Niger, data were collected between November 2023 and February 2024 from 3,292 households (98% response rate) and 3,840 women aged 15–49 (97% response rate). Finally, in Uganda, data were collected between September and October 2022 from 4,430 households (96% response rate) and 4,227 women aged 15–49 (96% response rate).

### Samples of facilities

All public facilities serving the survey EAs, including primary, secondary, and tertiary facilities (even if they were not within the EA), and up to three private facilities within each EA were invited to participate in the facility survey. If there were more than three private facilities per EA, three were randomly selected. Between September 2022 and February 2024, the facility survey was administered to 235 facilities in Burkina Faso (96% response rate), 218 facilities in Côte d’Ivoire (90% response rate), 291 facilities in Niger (95% response rate), and 382 facilities in Uganda (92% response rate). Pharmacies, chemist shops, and other facilities not expected to provide clinical infertility evaluation or treatment were excluded from analysis (*n* = 18 in Burkina Faso; *n* = 18 in Côte d’Ivoire; *n* = 34 in Niger; *n* = 68 in Uganda), as these outlets typically lack the clinical capacity to diagnose or manage infertility, even if they may serve as first points of contact for some reproductive health concerns; final analytic samples included *N* = 217 facilities in Burkina Faso, *N* = 200 in Côte d’Ivoire, *N* = 257 in Niger, and *N* = 314 in Uganda. Facility respondents included management staff or healthcare providers. Because PMA facility surveys are designed to capture facilities serving family planning needs, private fertility clinics operating independently of family planning services may be underrepresented, particularly in urban areas. Despite this, the sampling frame likely captures the majority of facilities where women in these settings would first seek infertility-related care.

All participating women and facility-based interviewees provided verbal (Burkina Faso, Côte d’Ivoire, Niger) or written (Uganda) informed consent to participate, documented in the ODK software. Ethical approval for the study protocol was provided by the Johns Hopkins Bloomberg School of Public Health institutional review board, Comité d’Ethique pour la recherche en santé and the Ministère de la Santé et Ministère de l’Enseignement Supérieur, de la Recherche Scientifique et de l’Innovation in Burkina Faso, Comité d’Ethique de la Recherche Institut Pasteur de la Côte d’Ivoire, the Comité Consultatif National d’Ethique in Niger, and the Makerere University School of Public Health Research and Ethics Committee in Uganda (IRB 14702).

### Measures

We grouped facilities by their sector (public/private) and level (primary/referral) for analysis. Public facilities are those managed by the government, while private facilities are those managed by NGOs, faith-based organizations, and other private entities. Primary and referral groupings varied by site, which should be considered when making cross-country comparisons of facility-level findings; in general, however, primary facilities included health centers and clinics, while referral facilities included hospitals and specialized facilities (i.e., surgery centers) (Appendix Table 1).

We also analyzed facility and female data by region, with modified groupings for Burkina Faso (due to small facility samples in some regions) and Côte d’Ivoire (due to the large number of regions). Burkina Faso was divided into three categories—1) Centre region, 2) Hauts-Bassin region, and 3) all other regions. Côte d’Ivoire was divided into 10 regions, aligning with the government’s geographic health divisions. In Niger and Uganda, all geographic regions were retained for analysis.

Facilities’ ability to provide infertility-related services was measured via one survey item: “Which of the following services are provided at this facility?” Possible response options included “STI testing” and “infertility services” (along with other services not examined in this study). An affirmative response to either option indicated availability at that facility (binary). We examined STI testing and infertility services separately. Of note, the facility survey captured STI testing availability only. STI treatment was not assessed; therefore, our measure reflects capacity for diagnosis but may not indicate comprehensive STI management capability. Additionally, the infertility services measure was a broad facility-reported measure of the availability of *any* service, which could range from basic counseling or diagnostic evaluation to referral or treatment; this measure therefore does not specifically capture ART availability, which represents only one component of a broader infertility care continuum, though ART has received the most attention in existing regional literature.

Among the female samples, we examined geographic access to infertility-related services using several sociodemographic and reproductive characteristics. These included age range in years (15–19, 20–29, 30–39, 40–49), education (none, primary, secondary, higher), marital status (currently married/cohabiting, divorced or separated/widowed, never married), wealth tertile (poorest, middle, wealthiest, generated via principal component analysis based on household assets, building materials, and water and sanitation sources collected from the household survey), residence (rural, urban), and parity (0, 1–2, 3–4, 5 + children).

We operationalized use of biomedical care (binary) from two survey items: “Have you ever sought help because you were having trouble getting pregnant?” If yes, “Who have you sought help from?” Help sought from hospitals, surgery centers, infertility clinics, family planning clinics, health centers/posts, maternity homes, mobile clinics, private doctors, and health outreach events was considered biomedical care, while help sought from fieldworkers, peer educators, community volunteers, community events, pharmacies/chemists, shops/markets, churches, religious leaders, friends/relatives, and traditional healers/herbalists was not considered biomedical care. Of note, pharmacies and chemists were not considered biomedical sources of care because it is unlikely that they are able to distribute effective infertility medications independent of formal sources of biomedical care (hospitals/clinics) where individuals would receive diagnoses and prescriptions.

### Analysis

All analyses were conducted separately by country. Using the facility data, we first examined the distribution of facilities by their characteristics (level, sector, region). We then estimated the proportion of facilities providing infertility-related services—specifically STI testing and infertility services—overall and by their characteristics. We then linked these facility data to female data using geospatial information (Appendix Fig. 1) to examine differences in geographic access to facilities offering STI testing and infertility services. Geographic access was measured using straight-line (Euclidean) distance between displaced EA centroid coordinates and facility GPS coordinates. EA-level centroids, rather than household-specific coordinates, were used as the unit of geographic reference, as PMA releases only aggregated, displaced centroid coordinates to protect respondent confidentiality. Geographic access analyses were conducted among all women aged 15–49 in the female survey sample, regardless of pregnancy history or reproductive intentions. This population-level approach provides a baseline measure of structural access independent of individual fertility desires, which may shift over time.

Using the linked facility-female data, we first estimated the proportion of women living within 5 km and 10 km from facilities offering STI testing and infertility services, respectively, overall and by women’s sociodemographic and reproductive characteristics. We tested for significant differences in proximity across women’s characteristics using design-based F-tests. We then fit bivariable and multivariable logistic regression models to examine the association between women’s characteristics and living within 5 km access and 10 km access to facilities offering STI testing and infertility services, respectively. Multivariable models adjusted for all of women’s characteristics (age, education, marital status, wealth, parity, and residence).

Lastly, among a sub-sample of women who reported they had ever sought care for difficulties conceiving, we fit bivariable and multivariable logistic regression models to examine the association between living within 5 km and 10 km of a facility offering STI testing and infertility services, respectively, and seeking biomedical care for difficulties conceiving. These models evaluate the *type* of care sought among women who had already sought any care for difficulties conceiving, rather than whether geographic access influences care-seeking itself. Multivariable models were adjusted for education and wealth—factors theoretically linked to biomedical care-seeking—while maintaining model parsimony. We selected 5 km (~ 3 miles) and 10 km (~ 6 miles) access thresholds for STI testing and infertility services, respectively—distances considered reasonably walkable—recognizing that STI testing is expected to be available at most health facilities across sub-Saharan Africa, while specialized infertility services require advanced training and are less likely to be available at nearby primary level facilities. These cutoffs are also broadly consistent with thresholds commonly used in geographic access research in the region [32–34]. We acknowledge, however, that they do not account for terrain, road quality, transport availability, or safety considerations, which may substantially affect actual travel time and access, particularly in rural and conflict-affected areas. Additionally, sensitivity analyses comparing 10 km and 20 km thresholds for infertility services yielded similar results; we present the 10 km findings, recognizing that women seeking specialized infertility care may need to travel farther than those seeking more widely available services such as STI testing. Fully adjusted models with all covariates (age, education, marital status, wealth, parity, and residence) were fit and yielded consistent results to models with only education and wealth; parsimonious models were retained given small subsample sizes in several countries, particularly Niger. All analyses were conducted in Stata version 19.5 [35], and analyses involving female data were weighted to account for the complex survey design, including probability of selection and nonresponse, and adjusted for clustering. Facility data are unweighted because the facility survey is designed to characterize facilities serving sampled EAs rather than to produce nationally representative facility-level estimates. As such, standard survey weighting procedures are not applicable.

## Results

### Facility characteristics

We present facility characteristics in Table 1. Across countries, most facilities were in the public sector, ranging from 82.5% in Uganda to 86.0% in Côte d’Ivoire, and provided primary-level care, ranging from 48.4% in Uganda to 79.0% in Niger. Within each country, there was substantial variation in the distribution of facilities by region (Appendix Table 2).

**Table 1:** Health facility characteristics, by country

	Burkina Faso		Côte d’Ivoire		Niger		Uganda	
	*N*	%	*N*	%	*N*	%	*N*	%
**Facility type**								
Public	182	83.9	172	86.0	215	83.7	259	82.5
Public primary	128	59.0	101	50.5	203	79.0	152	48.4
Public referral	54	24.9	71	35.5	12	4.7	107	34.1
Private	35	16.1	28	14.0	42	16.3	55	17.5
Total	217	100.0	200	100.0	257	100.0	314	100.0

Facility Ns and percentages are unweighted

### Facilities providing infertility-related services

Most facilities provided STI testing, ranging from 68.5% in Niger to 92.6% in Burkina Faso (Table 2). STI testing availability was substantially higher in public facilities than private facilities across countries, with a 15-percentage-point difference in Uganda to more than a 50-percentage-point difference in Côte d’Ivoire. More public referral facilities provided STI testing than public primary facilities in Uganda (99.1% vs. 76.3%), while the opposite was true in Niger (58.3% vs. 73.9%); STI testing was provided approximately equally by public referral and public primary facilities in Burkina Faso (98.2% vs. 97.7%) and Côte d’Ivoire (99.0% vs. 97.2%).

Across countries, far fewer facilities provided infertility services than STI testing, with less than half providing these services in Niger (28.4%), Uganda (30.9%), and Cote d’Ivoire (44.0%); 78.3% of facilities provided infertility services in Burkina Faso. Public facilities were more likely to provide infertility services compared to private facilities, with public referral facilities providing infertility services at higher proportions than public primary facilities across all countries (94.4% vs. 78.9% in Burkina Faso, 67.6% vs. 34.7% in Côte d’Ivoire, 66.7% vs. 27.6% in Niger, 56.1% vs. 16.5% in Uganda). Regional variation in service availability was substantial across countries: STI testing ranged from 87.3 to 95.5% across regions in Burkina Faso, 81.0–100.0% in Côte d’Ivoire, 50.0–85.7% in Niger, and 75.0–100.0% in Uganda; infertility service availability ranged from 78.0 to 79.6% in Burkina Faso, 28.4–86.7% in Côte d’Ivoire, 7.1–52.4% in Niger, and 10.0–53.3% in Uganda (Appendix Table 3).

**Table 2:** Health facilities offering STI testing and infertility services, overall and by facility type and country

		Burkina Faso			Côte d’Ivoire			Niger			Uganda		
			STI	Inf.		STI	Inf.		STI	Inf.		STI	Inf.
		*N*	%	%	*N*	%	%	*N*	%	%	*N*	%	%
**Facility type**													
Public		182	97.8	83.5	172	98.3	48.3	215	73.0	29.8	259	85.7	33.0
Public primary		128	97.7	78.9	101	99.0	34.7	203	73.9	27.6	152	76.3	16.5
Public referral		54	98.2	94.4	71	97.2	67.6	12	58.3	66.7	107	99.1	56.1
Private		35	65.7	51.4	28	46.4	17.9	42	45.2	21.4	55	70.9	21.8
Total		217	92.6	78.3	200	91.0	44.0	257	68.5	28.4	314	83.1	30.9

Facility Ns and percentages are unweighted; STI: sexually transmitted infection testing; Inf.: Infertility services

### Participant characteristics

Across countries, the majority of women were aged 20–29 (32.8% in Burkina Faso to 37.7% in Niger), had a primary level of education or less (59.0% in Uganda to 80.7% in Niger), and were married or cohabiting at the time of the survey (60.3% in Uganda to 78.4% in Niger) (Table 3). Most women in Burkina Faso, Niger, and Uganda lived in rural areas (78.1%, 82.1%, and 69.1%, respectively), while the majority in Côte d’Ivoire lived in urban areas (60.8%). Most women in Côte d’Ivoire and Uganda had one to two children (30.2% and 49.3%, respectively), while most women in Burkina Faso and Niger had five or more children at the time of the survey (30.9% and 36.8%, respectively).

**Table 3:** Percent of women aged 15–49 living within 5 km (km) of a facility offering STI testing and 10 km of a facility offering infertility services, overall and by characteristics and country

	Burkina Faso				Côte d’Ivoire				Niger				Uganda			
			STI 5 km	Inf. 10 km			STI 5 km	Inf. 10 km			STI 5 km	Inf. 10 km			STI 5 km	Inf. 10 km
	*N*	%	%	%	*N*	%	%	%	*N*	%	%	%	*N*	%	%	%
Age																
15–19	1270	20.4	64.2	61.0	647	18.3	**89.4**	76.8	946	22.6	46.4	26.2	887	20.9	73.0	59.4
20–29	2103	32.8	58.8	61.5	1249	35.5	**82.4**	70.8	1404	37.7	42.7	23.9	1549	37.6	78.1	64.4
30–39	1621	27.4	55.7	60.1	1050	27.1	**81.1**	68.0	922	24.8	39.2	20.6	1140	26.9	76.2	61.2
40–49	1084	19.4	58.3	58.6	688	19.2	**79.8**	70.7	568	14.9	41.1	23.2	651	14.6	72.5	55.6
Education																
None	2509	56.4	**47.4**	**51.9**	1458	40.2	**74.4**	**65.9**	1771	64.1	**31.1**	**14.9**	236	4.1	**71.1**	**39.3**
Primary	1247	18.7	**66.2**	**63.6**	901	23.6	**80.6**	**67.4**	670	16.6	**48.9**	**24.0**	2488	54.9	**68.7**	**52.2**
Secondary	2053	23.0	**78.3**	**75.6**	1049	30.0	**92.5**	**76.4**	1127	17.1	**72.1**	**46.6**	1194	31.3	**83.6**	**71.7**
Higher	269	1.9	**97.5**	**97.0**	225	6.2	**99.3**	**93.2**	271	2.2	**95.9**	**90.2**	308	9.7	**92.0**	**88.1**
Marital status																
Married/cohabiting	4037	74.4	**55.1**	**57.8**	2256	62.1	**76.7**	**66.5**	2583	78.4	**37.7**	**19.5**	2555	60.3	74.3	**59.4**
Divorced or separated/widowed	288	3.7	**63.2**	**65.9**	183	4.5	**78.6**	**64.2**	208	3.7	**58.9**	**31.8**	708	15.5	75.8	**58.4**
Never married	1753	21.9	**71.2**	**68.4**	1195	33.4	**94.8**	**80.6**	1049	17.9	**59.9**	**39.3**	963	24.2	79.1	**67.5**
Wealth tertile																
Poorest	1228	37.5	**45.3**	**49.2**	1241	29.9	**53.8**	**48.7**	729	31.4	**21.0**	**9.8**	1560	31.2	**58.9**	**37.9**
Middle	1333	31.8	**50.9**	**52.1**	1214	33.1	**90.8**	**66.1**	730	33.0	**30.9**	**8.3**	1431	32.6	**74.7**	**58.3**
Wealthiest	3517	30.7	**84.0**	**82.6**	1179	37.0	**99.2**	**93.7**	2380	35.6	**72.1**	**49.6**	1236	36.3	**91.0**	**83.9**
Parity																
0	1786	23.7	**69.9**	**68.2**	937	27.0	**94.1**	**82.4**	1252	24.5	**50.8**	**33.1**	1003	24.9	**80.0**	**68.0**
1–2	1605	23.5	**63.5**	**65.1**	1096	30.2	**83.7**	**68.5**	751	19.6	**44.6**	**23.3**	1226	28.9	**77.5**	**64.4**
3–4	1341	21.9	**61.4**	**63.7**	781	21.8	**80.3**	**67.9**	701	19.1	**45.2**	**22.9**	851	20.4	**78.4**	**62.9**
5+	1346	30.9	**45.5**	**48.6**	820	21.0	**69.7**	**63.7**	1136	36.8	**34.3**	**17.5**	1146	25.8	**67.4**	**49.9**
Residence																
Rural	2542	78.1	**48.5**	**50.1**	1479	39.2	**57.0**	**47.4**	1823	82.1	**30.1**	**8.4**	2759	69.1	**66.7**	**49.3**
Urban	3536	21.9	**96.2**	**97.3**	2155	60.8	**99.5**	**86.4**	2016	17.9	**98.9**	**92.4**	1468	30.9	**95.7**	**87.9**
**Total**	6078	100.0	59.0	60.4	3634	100.0	82.8	71.1	3840	100.0	42.5	23.5	4227	100.0	75.7	61.2

Female Ns are unweighted, percentages are weighted; bold indicates p-value < 0.05 from design-based F test; STI: sexually transmitted infection testing; Inf.: Infertility services

### Access to infertility-related services among women of reproductive age

Patterns in geographic access to infertility-related services were similar across countries (Table 3). Women with lower levels of formal education and wealth, women with more children, and women living in rural areas were significantly less likely to live within 5 km of a facility providing STI testing and within 10 km of a facility providing infertility services compared to their formally educated, wealthier, lower parity, and urban counterparts (*p* < 0.05). In Burkina Faso, Côte d’Ivoire, and Niger, married/cohabiting women were significantly less likely to live within 5 km of a facility providing STI testing, and across all sites, were significantly less likely to live within 10 km of a facility providing infertility services, compared to those divorced/widowed or never married. In Côte d’Ivoire alone, older women were significantly less likely to live within 5 km of a facility offering STI testing compared to younger women. Regional disparities in geographic proximity were similarly pronounced: the proportion of women living within 5 km of STI testing ranged from 51.7 to 100.0% across regions in Burkina Faso, 53.7–100.0% in Côte d’Ivoire, 14.1–100.0% in Niger, and 36.0–100.0% in Uganda; the proportion living within 10 km of infertility services ranged from 52.7 to 100.0% in Burkina Faso, 0.0–96.3% in Côte d’Ivoire, 0.0–100.0% in Niger, and 0.0–100.0% in Uganda (Appendix Table 4).

The independent odds of living close to a facility offering STI testing or infertility services varied by women’s characteristics (Table 4). Across countries, rural residence was the strongest and most consistent predictor of reduced geographic proximity to both services. Age was positively associated with geographic proximity to infertility services in Burkina Faso (aOR 1.73, 95% CI 1.00-2.98). Education was positively associated with geographic proximity to services in Burkina Faso and Uganda—women with at least a secondary level of education had increased odds of living near STI testing in Burkina Faso (aOR 2.13, 95% CI 1.31–3.44) and near infertility services in Burkina Faso (aOR 1.64, 95% CI 1.04–2.58) and Uganda (aOR 1.36, 95% CI 1.01–1.82). Married/cohabiting women had decreased odds of geographic proximity to STI testing in Côte d’Ivoire (aOR 0.50, 95% CI 0.29–0.87) and Niger (aOR 0.48, 95% CI 0.32–0.71). Wealth was positively associated with an increased odds of geographic proximity to a facility offering STI testing in Côte d’Ivoire, Niger, and Uganda (ref. poorest, aORs for wealthiest tertile ranging from 3.16, 95% CI 1.28–7.76 in Uganda to 10.19, 95% CI 1.79–57.95 in Côte d’Ivoire) and to a facility offering infertility services in Côte d’Ivoire and Uganda (aORs ranging from 1.80, 95% CI 1.04–3.10 for middle tertile in Uganda to 6.52, 95% CI 2.27–18.78 for wealthiest tertile in Côte d’Ivoire). Parity was inconsistently associated with proximity across countries.

**Table 4:** Odds of living within 5 km (km) of a facility offering STI testing and 10 km of a facility offering infertility services among women aged 15–49, by country

	Burkina Faso (*n* = 6,070)		Côte d’Ivoire (*n* = 3,631)		Niger (*n* = 3,834)		Uganda (*n* = 4,227)	
	STI 5 km	Inf. 10 km	STI 5 km	Inf. 10 km	STI 5 km	Inf. 10 km	STI 5 km	Inf. 10 km
	aOR (95% CI)							
Age (ref. 15–19)								
20–29	0.98 (0.61–1.57)	1.37 (0.84–2.23)	1.07 (0.72–1.61)	1.07 (0.77–1.50)	0.91 (0.58–1.43)	1.08 (0.66–1.78)	1.29 (0.91–1.84)	1.12 (0.80–1.56)
30–39	1.17 (0.65–2.11)	**1.65** **(0.92–2.93)**	**1.75** **(0.97–3.18)**	1.22 (0.78–1.90)	0.87 (0.52–1.47)	0.81 (0.31–2.08)	**1.38** **(0.96-2.00)**	1.18 (0.81–1.72)
40–49	1.53 (0.87–2.68)	**1.73** **(1.00-2.98)** ^*^	1.42 (0.80–2.53)	1.35 (0.84–2.15)	0.98 (0.58–1.67)	1.01 (0.49–2.09)	1.32 (0.91–1.92)	1.10 (0.76–1.58)
Education (ref. None/primary)								
Secondary/Higher	**2.13** **(1.31–3.44)** ^**^	**1.64** (1.04–2.58)_*_	1.26 (0.69–2.28)	0.84 (0.56–1.27)	1.91 (0.85–4.29)	0.95 (0.50–1.81)	1.29 (0.89–1.88)	**1.36** **(1.01–1.82)** ^*^
Married/cohabiting (ref. No)								
Yes	1.11 (0.65–1.91)	1.18 (0.78–1.77)	**0.50** **(0.29–0.87)** ^*^	0.89 (0.61–1.30)	**0.48** **(0.32–0.71)** ^***^	0.99 (0.56–1.77)	1.01 (0.75–1.36)	1.09 (0.85–1.42)
Wealth tertile (ref. Poorest)								
Middle	1.23 (0.77–1.96)	1.06 (0.68–1.66)	**3.75** **(1.65–8.52)** ^**^	1.26 (0.60–2.65)	1.59 (0.90–2.82)	0.67 (0.37–1.23)	1.62 (0.90–2.92)	**1.80** **(1.04–3.10)** ^*^
Wealthiest	2.18 (0.83–5.75)	1.47 (0.58–3.73)	**10.19** **(1.79–57.95)** ^**^	**6.52** **(2.27–18.78)** ^**^	**3.30** **(1.33–8.21)** ^*^	1.24 (0.52–2.97)	**3.16** **(1.28–7.76)** ^*^	**4.11** **(1.90–8.92)** ^***^
Parity (ref. 0)								
1+	0.71 (0.44–1.14)	**0.63** **(0.40–0.99)** ^*^	0.78 (0.45–1.33)	**0.72** **(0.49–1.05)**	**2.00** **(1.33–3.02)** ^**^	1.15 (0.59–2.22)	0.74 (0.50–1.09)	**0.75** **(0.54–1.04)**
Residence (ref. Urban)								
Rural	**0.07** **(0.02–0.25)** ^***^	**0.04** **(0.01–0.16)** ^***^	**0.02** **(0.00-0.13)** ^***^	**0.30** **(0.10–0.92)** ^*^	**0.01** **(0.00-0.12)** ^***^	**0.01** **(0.00-0.05)** ^***^	**0.15** **(0.04–0.57)** ^**^	**0.24** **(0.09–0.69)** ^**^

Estimates are weighted; bold indicates p-value < 0.1; *<0.05; **<0.01; ***<0.001. STI: sexually transmitted infection testing; Inf.: Infertility services

### Use of infertility-related services among women of reproductive age

Few women had ever sought care for difficulties conceiving across countries: 8.0% (*n* = 505) in Burkina Faso, 15.0% (*n* = 436) in Côte d’Ivoire, 2.6% (*n* = 109) in Niger, and 5.9% (*n* = 226) in Uganda (Table 5). Of these women, 52.2%, 52.6%, 70.7%, and 64.1%, respectively, reported seeking biomedical care. Proximity to infertility-related services was associated with biomedical care-seeking in two of four countries. In Burkina Faso, women living within 5 km of a facility offering STI testing had approximately three times the odds of seeking biomedical care for difficulties conceiving compared to those living farther away (aOR 2.94, 95% CI 1.48–5.82) (Table 6). Similarly, in Niger, women living within 5 km and 10 km of a facility offering STI testing and infertility services, respectively, had higher odds of seeking biomedical care (aOR_STI_ 7.19, 95% CI 1.23–42.02; aOR_INF_ 8.46, 95% CI 1.48–48.36) than those living farther away. Proximity to a facility offering infertility-related services was not associated with biomedical care-seeking in Côte d’Ivoire or Uganda.

**Table 5:** Infertility related help-seeking among women aged 15–49, by country

		Burkina Faso		Côte d’Ivoire		Niger		Uganda	
		*n*	%	*n*	%	*n*	%	*n*	%
Ever sought help for difficulties getting pregnant		505	8.0	436	15.0	109	2.6	226	5.9
Ever sought biomedical help for difficulties getting pregnant^**±**^		324	52.2	190	52.6	90	70.7	152	64.1

Female Ns are unweighted, percentages are weighted

^**±**^Among those who sought any help

**Table 6:** Odds of seeking biomedical care for difficulties getting pregnant if living within 5 km (km) of facility offering STI testing and 10 km of facility offering infertility services, among women aged 15–49 who ever sought care for difficulties getting pregnant, by country

		Burkina Faso (*n* = 505)		Cote d’Ivoire (*n* = 436)		Niger (*n* = 109)		Uganda (*n* = 226)	
		OR (95% CI)	aOR (95% CI)	OR (95% CI)	aOR (95% CI)	OR (95% CI)	aOR (95% CI)	OR (95% CI)	aOR (95% CI)
Live within 5 km of facility offering STI testing (ref. No)		**3.32** **(1.80–6.14)** ^***^	**2.94** **(1.48–5.82)** ^**^	1.20 (0.65–2.23)	0.59 (0.25–1.39)	**4.46** **(1.48–13.44)** ^**^	**7.19** **(1.23–42.02)** ^*^	1.32 (0.53–3.29)	1.18 (0.47–2.93)
Live within 10 km of facility offering inf. services (ref. No)		**1.74** **(0.95–3.20)**	1.36 (0.71–2.59)	**1.59** **(0.99–2.56)**	0.98 (0.55–1.74)	**4.72** **(1.57–14.20)** ^**^	**8.46** **(1.48–48.36)** ^*^	1.42 (0.54–3.70)	1.23 (0.47–3.24)

Multivariable models adjusted for education and wealth tertile

Estimates are weighted; bold indicates p-value < 0.1; *<0.05; **<0.01; ***<0.001

## Discussion

Our results indicate that availability of and geographic access to infertility-related services within four sub-Saharan African countries remain limited and are inequitably accessible. Although the availability of STI testing was generally high across countries, infertility services remain largely unavailable, with notable disparities in geographic proximity to both STI testing and infertility services based on women’s characteristics. Additionally, among women who sought help for difficulties conceiving, proximity to a facility offering infertility-related services was associated with increased odds of seeking biomedical care in Burkina Faso and Niger.

Our findings of moderate to widespread coverage of STI testing availability—ranging from 68.5% in Niger to 92.6% in Burkina Faso—align with the WHO’s 2022–2030 strategic actions for scaling up primary prevention and access to screening for STIs in all levels of healthcare facilities [36]; however, they reveal gaps in comprehensive care, with notable variation between countries and facility types, likely reflecting varying health system capacities and policy landscapes. Of note, it is unclear what STI tests are available within these facilities. STI testing in resource-constrained settings is often limited to available point-of-care rapid diagnostic tests, such as those for syphilis or HIV, and may not include tests for other common STIs—such as chlamydia, gonorrhea, *Trichomonas vaginalis* and *Mycoplasma genitalium*—which are more costly and require laboratory-based testing [37, 38]. Additionally, testing and/or diagnosis in resource-constrained settings is often based on presentation of symptoms, which is inadequate for STIs that are commonly asymptotic, such as chlamydia and gonorrhea [39]. Asymptomatic STIs often go untreated but can result in long-term complications, such as pelvic inflammatory disease, potentially resulting in subsequent tubal factor infertility [16]. Untreated asymptomatic STIs are a critical driver of preventable infertility [17], underscoring the necessity of widespread STI testing and treatment. The availability of testing alone does not necessarily indicate usage or subsequent treatment. Previous studies in the region have highlighted that barriers to available STI testing and treatment persist, including low levels of health literacy, difficulties with treatment of both partners, and stigmatizing behavior from health care professionals [22, 40].

Overall, provision of infertility services across sites was inadequate. While the majority of facilities reported offering infertility services in Burkina Faso (78.3%), fewer than half do so in Côte d’Ivoire (44.0%), Niger (28.4%), and Uganda (30.9%), providing limited opportunity for couples struggling to conceive to receive necessary care. Although few studies have investigated the provision of infertility-related services at healthcare facilities in sub-Saharan Africa, our findings align with existing research in the region, which highlights significant service gaps. For example, only 66% of facilities in the Gambia offer any infertility-related services [30], and Africa accounts for less than 2% of ART coverage globally [41]. We also found disparities in geographic access to health facilities offering infertility-related services, with those experiencing the greatest structural disadvantages least likely to live within 10 km of a facility providing infertility-related services. This is consistent with findings in relation to other health services [32–34, 42], potentially compounding social disparities in health outcomes and achievement of one’s reproductive goals.

Most women who sought any care for difficulties conceiving sought biomedical care specifically (50–70%). Proximity to a facility offering infertility-related services was associated with nearly three times the odds of seeking biomedical care in Burkina Faso and more than eight times the odds in Niger. Previous studies in the region, including in Nigeria, the Gambia, Uganda, and Rwanda, have found delayed or low levels of biomedical care seeking for difficulties conceiving, with many women relying on traditional remedies before seeking facility-based care [26, 43–46]. Our findings suggest geographic proximity to services may at least partially explain the use of biomedical infertility services in some contexts; this is consistent with existing literature regarding the use of recommended or facility-based care for other reproductive services [47, 48].

These findings have several important implications for policy and practice. Building on the demonstrated importance of policy alignment and strong political support for fertility care [49–51], countries facing competing health priorities have an opportunity to recognize and address the burden of infertility as part of a broader reproductive health agenda; several sub-Saharan African countries have begun to do so by developing or approving national infertility guidelines [52, 53]. The newly released WHO infertility guidelines also provide a timely framework to support this work [54]. Additionally, the availability of STI testing infrastructure presents an opportunity to leverage existing efforts to integrate infertility prevention services. However, not all facilities can or should be expected to offer infertility treatment, which requires specialized training, laboratory capacity, and equipment. Countries should consider developing clinical protocols that link STI screening with targeted fertility counseling and basic infertility evaluation in family planning and pre-conception care settings, where couples may be explicitly expressing reproductive intentions. The geographic disparities we identified call for targeted facility and outreach strengthening potentially through telemedicine, task-shifting to lower-level providers, and mobile ART laboratories such as the Walking Egg Project (a low-cost simplified IVF culture system) [33, 55, 56], which can bring care directly to underserved populations. The gaps in infertility service availability—particularly in Niger, Côte d’Ivoire, and Uganda, and in peripheral and rural regions within these countries—suggest a need for geographically targeted expansion of services, expanded insurance coverage to increase demand and support service expansion, and critically, clear and functional referral pathways to higher-level care.

This study has several limitations. Sampling was restricted to facilities likely to provide family planning services, given the objectives of the broader PMA project, and may underestimate the true availability of infertility-related services, particularly in urban areas where private fertility clinics may operate independently of family planning services. The facility survey’s measure of “infertility services” lacks specificity, limiting our understanding of the types of services provided, which could range from consultations and fertility counseling to diagnostic testing and treatment options such as IVF. Additionally, our measure of biomedical care-seeking encompasses a wide range of providers—from community health centers to specialized infertility clinics—and therefore does not distinguish receipt of basic care from specialized infertility evaluation or treatment. This likely means that biomedical care-seeking as measured here overestimates receipt of specialized infertility care, and that observed associations between proximity and care-seeking may partly reflect access to general health services rather than dedicated infertility services. We were unable to examine biomedical care-seeking by provider level given the small subsample sizes. The facility survey also only inquired about STI testing, with no additional questions on specific types of tests or availability of STI treatment, both of which are essential for effective STI management, and there was no assessment of STI testing occurring outside of health facilities.

Given limitations in survey measures, we were unable to assess indicators of quality of care (i.e., provider training, equipment functionality, patient satisfaction), which can vary dramatically between facilities and significantly impact treatment outcomes. Service interruptions (i.e., supply chain issues, equipment failures, staff shortages), which are common challenges in resource-limited settings, are not captured in our cross-sectional assessment but can substantially affect service accessibility. This is particularly salient in the Sahel region (Burkina Faso and Niger), where ongoing conflict and humanitarian crises have displaced large populations and threaten to erode service gains reflected in our data; future research should examine how humanitarian crises affect infertility service availability and access in this context [57]. Geographic proximity at the time of survey reflects current service availability and may not correspond to availability when women previously sought care for difficulties conceiving, which could have occurred years earlier. This temporal mismatch may attenuate or distort observed associations between proximity and care-seeking. We also lack information on service costs, user fees, and insurance coverage, factors that represent major barriers to infertility care-seeking in sub-Saharan Africa [58] and can render geographically proximate services functionally inaccessible to many women. Unmeasured psychosocial and sociocultural factors represent important areas for future research and may modify the relationship between geographic access and care-seeking. PMA GPS coordinates are subject to random displacement, up to 2 km for urban EAs and up to 5 km for rural EAs, with 1% of rural EAs displaced up to 10 km. Because our proximity thresholds (5 km and 10 km) are of similar magnitude to the maximum displacement distances, particularly in rural areas, some misclassification of women as near or far from facilities is possible, potentially underestimating true disparities in proximity to services. This study relied exclusively on female survey data and facility-level reports, and therefore cannot capture male factor infertility or the experiences of women outside the 15–49 age range. A complete picture of infertility care needs in these settings would require couple-level data or surveys including male partners. Finally, results from the multivariable models in Niger should be interpreted with caution given the small analytic subsample (*n* = 109), which yielded measures with considerable uncertainty.

This study has a number of strengths. It links nationally representative, population-based female survey data with health facility data across four countries using standardized PMA methods and instruments, enabling rigorous cross-country comparisons while minimizing methodological heterogeneity. The geospatial linkage of women’s EA locations to facility GPS coordinates allows for direct examination of disparities in geographic access to care—a methodological approach not yet applied to infertility services in sub-Saharan Africa. Overall, the study addresses a significant gap in the literature by providing baseline data on the availability, geographic accessibility, and use of infertility services across four sub-Saharan African countries, a critical yet often overlooked area of reproductive health.

## Conclusion

Our findings reveal both opportunities to build upon existing STI testing infrastructure and critical gaps in specialized infertility care that require coordinated efforts to ensure comprehensive, accessible, and equitable reproductive health services. Future research incorporating detailed service inventories, quality assessments, cost analyses, and longitudinal tracking of service availability would provide a more comprehensive understanding of infertility care accessibility and inform targeted interventions to improve achievement of reproductive goals in sub-Saharan Africa. Governments and NGOs should establish infertility-related guidelines and prioritize investments in infertility services, including STI prevention and treatment, reproductive health counseling, and where feasible, more advanced services such as ART, to prevent and treat infertility.

## Supplementary Information

Below is the link to the electronic supplementary material.


Supplementary Material 1


## Data Availability

Data are publicly available on the Johns Hopkins Research Data Repository (https://archive.data.jhu.edu/dataverse/pma).

## Abbreviations

aOR: Adjusted odds ratio
ART: Assisted Reproductive Technology
CI: Confidence interval
ICSI: Intracytoplasmic sperm injection
IUI: Intrauterine insemination
IVF: In vitro fertilization
km: Kilometers
ODK: Open Data Kit
PMA: Performance Monitoring for Action
STIs: Sexually transmitted infections
WHO: World Health Organization
