# Supplementary material for: Infertility service availability, geographic accessibility, and use: results from linked facility-female cross-sectional data in Burkina Faso, Côte d’Ivoire, Niger, and Uganda
Source: BMC Health Serv Res. 2026 Aug 4;26:1219. doi: 10.1186/s12913-026-15214-6 (PMC13536680; doi:10.1186/s12913-026-15214-6)
Supplement: Supplementary file 1 [file 12913_2026_15214_MOESM1_ESM.docx]

| **Appendix Table 1. Primary and referral facility groupings, by country** | | |
| --- | --- | --- |
|  | **Primary facilities** | **Referral facilities** |
| **Burkina Faso** | Health center  Private health center | Private clinic  Regional hospital  Surgery center  Teaching hospital |
| **Côte d’Ivoire** | Urban health center  Health center  Rural health center  Rural maternity  Clinic  Private infirmary  Private maternity  Med social center  Religious center | Institute  Teaching hospital  Regional hospital  General hospital |
| **Niger** | Regional hospital  District hospital  Integrated health center  Health hut  Polyclinic or private clinic  Private treatment room  Care office/medical office | General referral hospital  National hospital  Mother and child center |
| **Uganda** | Health center 3  Health center 2  Health clinic | Hospital  Health center 4 |

**Appendix Figure 1. Sample flow diagrams, by country**

| **Appendix Table 2. Health facilities by country and region** | | |
| --- | --- | --- |
|  | **N** | **%** |
| **Burkina Faso** |  |  |
| Centre | 55 | 25.4 |
| Hauts-Bassin | 44 | 20.3 |
| All other regions | 118 | 54.4 |
| Total | 217 | 100.0 |
| **Cote d'Ivoire** |  |  |
| Abengourou | 8 | 4.0 |
| Abidjan | 74 | 37.0 |
| Bondoukou | 8 | 4.0 |
| Bouake | 15 | 7.5 |
| Daloa | 18 | 9.0 |
| Korhogo | 10 | 5.0 |
| Man | 14 | 7.0 |
| Odienne | 2 | 1.0 |
| San Pedro | 21 | 10.5 |
| Yamoussoukro | 30 | 15.0 |
| Total | 200 | 100.0 |
| **Niger** |  |  |
| Agadez | 14 | 5.5 |
| Diffa | 10 | 3.9 |
| Dosso | 28 | 10.9 |
| Maradi | 29 | 11.3 |
| Niamey | 101 | 39.3 |
| Tahoua | 26 | 10.1 |
| Tillaberi | 21 | 8.2 |
| Zinder | 28 | 10.9 |
| Total | 257 | 100.0 |
| **Uganda** |  |  |
| Acholi | 19 | 6.1 |
| Ankole | 38 | 12.1 |
| Bukedi | 11 | 3.5 |
| Bunyoro | 30 | 9.6 |
| Busoga | 29 | 9.2 |
| Elgon | 19 | 6.1 |
| Kampala | 13 | 4.1 |
| Karamoja | 10 | 3.2 |
| Kigezi | 20 | 6.4 |
| Lango | 18 | 5.7 |
| North Buganda | 24 | 7.6 |
| South Buganda | 40 | 12.7 |
| Testo | 10 | 3.2 |
| Tooro | 18 | 5.7 |
| West Nile | 15 | 4.8 |
| Total | 314 | 100.0 |

Ns and percentages are unweighted

| **Appendix Table 3. Health facilities offering STI testing and infertility services, by country and region** | | | |
| --- | --- | --- | --- |
|  |  | **STI**  **testing** | **Infertility**  **services** |
|  | **N** | **%** | **%** |
| **Burkina Faso** |  |  |  |
| Centre | 55 | 87.3 | 78.2 |
| Hauts-Bassin | 44 | 95.5 | 79.6 |
| All other regions | 118 | 94.1 | 78.0 |
| Total | 217 | 92.6 | 78.3 |
| **Cote d'Ivoire** |  |  |  |
| Abengourou | 8 | 87.5 | 62.5 |
| Abidjan | 74 | 89.2 | 28.4 |
| Bondoukou | 8 | 87.5 | 75.0 |
| Bouake | 15 | 100.0 | 86.7 |
| Daloa | 18 | 100.0 | 44.4 |
| Korhogo | 10 | 90.0 | 60.0 |
| Man | 14 | 92.9 | 57.1 |
| Odienne | 2 | 100.0 | 50.0 |
| San Pedro | 21 | 81.0 | 28.6 |
| Yamoussoukro | 30 | 93.3 | 46.7 |
| Total | 200 | 91.0 | 44.0 |
| **Niger** |  |  |  |
| Agadez | 14 | 71.4 | 7.1 |
| Diffa | 10 | 80.0 | 30.0 |
| Dosso | 28 | 50.0 | 21.4 |
| Maradi | 29 | 82.8 | 24.1 |
| Niamey | 101 | 64.4 | 28.7 |
| Tahoua | 26 | 76.9 | 26.9 |
| Tillaberi | 21 | 85.7 | 52.4 |
| Zinder | 28 | 60.7 | 32.1 |
| Total | 257 | 68.5 | 28.4 |
| **Uganda** |  |  |  |
| Acholi | 19 | 84.2 | 36.8 |
| Ankole | 38 | 76.3 | 15.8 |
| Bukedi | 11 | 90.9 | 27.3 |
| Bunyoro | 30 | 80.0 | 30.0 |
| Busoga | 29 | 75.9 | 41.4 |
| Elgon | 19 | 89.5 | 21.1 |
| Kampala | 13 | 92.3 | 38.5 |
| Karamoja | 10 | 80.0 | 10.0 |
| Kigezi | 20 | 75.0 | 30.0 |
| Lango | 18 | 77.8 | 44.4 |
| North Buganda | 24 | 91.7 | 29.2 |
| South Buganda | 40 | 82.5 | 45.0 |
| Testo | 10 | 90.0 | 10.0 |
| Tooro | 18 | 83.3 | 11.1 |
| West Nile | 15 | 100.0 | 53.3 |
| Total | 314 | 83.1 | 30.9 |

Ns and percentages are unweighted

| **Appendix Table 4. Percent of women aged 15-49 living within 5 kilometers (km) of a facility offering STI testing and 10 km of a facility offering infertility services, by country and region** | | | | |
| --- | --- | --- | --- | --- |
|  |  |  | **STI**  **5 km** | **Inf.**  **10 km** |
|  | **N** | **%** | **%** | **%** |
| **Burkina Faso** |  |  |  |  |
| Centre | 1489 | 10.8 | **100.0** | **100.0** |
| Hauts-Bassin | 1502 | 10.5 | **71.3** | **77.4** |
| All other regions | 3087 | 78.7 | **51.7** | **52.7** |
| Total | 6078 | 100.0 | 59.0 | 60.4 |
| **Cote d'Ivoire** |  |  |  |  |
| Abengourou | 177 | 5.5 | 83.3 | **53.4** |
| Abidjan | 1480 | 44.8 | 96.4 | **84.1** |
| Bondoukou | 100 | 5.1 | 53.7 | **40.6** |
| Bouake | 254 | 8.6 | 82.6 | **96.3** |
| Daloa | 336 | 7.7 | 62.3 | **56.0** |
| Korhogo | 212 | 5.1 | 71.9 | **70.3** |
| Man | 278 | 5.1 | 69.1 | **55.1** |
| Odienne | 47 | 1.3 | 100.0 | **0.0** |
| San Pedro | 307 | 5.1 | 59.6 | **45.3** |
| Yamoussoukro | 443 | 11.7 | 75.8 | **61.2** |
| Total | 3634 | 100.0 | 82.8 | 71.1 |
| **Niger** |  |  |  |  |
| Agadez | 100 | 1.5 | **100.0** | **0.0** |
| Diffa | 102 | 2.1 | **64.5** | **36.8** |
| Dosso | 323 | 13.7 | **14.1** | **16.1** |
| Maradi | 567 | 22.8 | **53.8** | **24.1** |
| Niamey | 1294 | 7.5 | **100.0** | **100.0** |
| Tahoua | 564 | 19.6 | **31.1** | **9.6** |
| Tillaberi | 248 | 12.5 | **32.5** | **17.2** |
| Zinder | 642 | 20.4 | **38.2** | **17.0** |
| Total | 3840 | 100.0 | 42.4 | 23.5 |
| **Uganda** |  |  |  |  |
| Acholi | 245 | 6.4 | **73.4** | **71.9** |
| Ankole | 349 | 5.9 | **86.1** | **32.7** |
| Bukedi | 178 | 2.7 | **75.7** | **24.8** |
| Bunyoro | 401 | 6.6 | **37.6** | **44.8** |
| Busoga | 279 | 13.2 | **77.3** | **80.4** |
| Elgon | 264 | 5.5 | **100.0** | **81.0** |
| Kampala | 236 | 9.0 | **100.0** | **100.0** |
| Karamoja | 153 | 1.8 | **48.5** | **6.2** |
| Kigezi | 158 | 2.3 | **97.6** | **81.1** |
| Lango | 311 | 4.4 | **52.2** | **57.1** |
| North Buganda | 358 | 10.6 | **70.4** | **63.1** |
| South Buganda | 494 | 14.9 | **93.2** | **87.1** |
| Testo | 200 | 4.9 | **36.0** | **0.5** |
| Tooro | 396 | 8.3 | **59.3** | **0.0** |
| West Nile | 205 | 3.6 | **91.9** | **80.1** |
| Total | 4227 | 100.0 | 75.7 | 61.2 |

Ns are unweighted, percentages are weighted

STI: sexually transmitted infection testing; Inf.: Infertility services
